# Research priorities for child and adolescent physical activity and sedentary behaviours: an international perspective using a twin-panel Delphi procedure

**DOI:** 10.1186/1479-5868-10-112

**Published:** 2013-10-24

**Authors:** Lauren Gillis, Grant Tomkinson, Timothy Olds, Carla Moreira, Candice Christie, Claudio Nigg, Ester Cerin, Esther Van Sluijs, Gareth Stratton, Ian Janssen, Jeremy Dorovolomo, John J Reilly, Jorge Mota, Kashef Zayed, Kent Kawalski, Lars Bo Andersen, Manuel Carrizosa, Mark Tremblay, Michael Chia, Mike Hamlin, Non Eleri Thomas, Ralph Maddison, Stuart Biddle, Trish Gorely, Vincent Onywera, Willem Van Mechelen

**Affiliations:** 1 Health and Use of Time (HUT) Group, University of South Australia, Adelaide, South Australia, Australia; 2 Research Centre in Physical Activity, Health and Leisure, Faculty of Sport, University of Porto, Porto, Portugal; 3 Ergonomics Unit, Rhodes University, Grahamstown, South Africa; 4 Department of Public Health Sciences, University of Hawaii at Manoa, Honolulu, HI, USA; 5 Institute of Human Performance, The University of Hong Kong, Hong Kong, China; 6 MRC Epidemiology Unit, Institute of Metabolic Science, Addenbrooke’s Hospital, Cambridge, UK; 7 The Research Institute for Sport and Exercise Sciences, Liverpool John Moores University, Liverpool, UK; 8 School of Physical and Health Education, Queen's University, Kingston, Ontario, Canada; 9 University of the South Pacific, Laucala Campus, Suva, Fiji Islands; 10 Physical Activity for Health Research Group, School of Psychological Sciences and Health, University of Strathclyde, Jordanhill, Glasgow, UK; 11 Department of Physical Education, Sultan Qaboos University, Muscat, Sultanate of Oman; 12 Physical and Health Education and Department of Psychology, School of Exercise Science, University of Victoria, Victoria, BC, Canada; 13 Center for Research in Childhood Health, Institute of Sport Sciences and Clinical Biomechanics, University of Southern Denmark, Odense 5230, Denmark; 14 Education Faculty, University of Extremadura, Avda de Elvas s/n, Badajoz, Spain; 15 Healthy Active Living and Obesity Research Group, Children's Hospital of Eastern Ontario Research Institute, Ottawa, Canada; 16 Physical Education & Sports Science, National Institute of Education, Nanyang Technological University, Singapore, Singapore; 17 Department of Social Science, Parks, Recreation, Tourism and Sport, Lincoln University, Christchurch, New Zealand; 18 Centre for Children and Young People's Health and Well-Being, School of Human and Health Sciences, Swansea University, Swansea, UK; 19 Clinical Trials Research Unit, School of Population Health, University of Auckland, Auckland 1142, New Zealand; 20 School of Sport, Exercise & Health Sciences, Loughborough University, Loughborough, Leicestershire, UK; 21 Institute of Youth Sport, School of Sport and Exercise Sciences, Loughborough University, Loughborough, LE 11 3TU, UK; 22 Department of Exercise, Kenyatta University, Recreation and Sport Science, Nairobi, Kenya; 23 Department of Public & Occupational Health, EMGO Institute, VU University Medical Center, Amsterdam, The Netherlands

**Keywords:** Physical activity, Sedentary behaviour, Research priorities, Children, Adolescents

## Abstract

**Background:**

The quantity and quality of studies in child and adolescent physical activity and sedentary behaviour have rapidly increased, but research directions are often pursued in a reactive and uncoordinated manner.

**Aim:**

To arrive at an international consensus on research priorities in the area of child and adolescent physical activity and sedentary behaviour.

**Methods:**

Two independent panels, each consisting of 12 experts, undertook three rounds of a Delphi methodology. The Delphi methodology required experts to anonymously answer questions put forward by the researchers with feedback provided between each round.

**Results:**

The primary outcome of the study was a ranked set of 29 research priorities that aimed to be applicable for the next 10 years. The top three ranked priorities were: developing effective and sustainable interventions to increase children’s physical activity long-term; policy and/or environmental change and their influence on children’s physical activity and sedentary behaviour; and prospective, longitudinal studies of the independent effects of physical activity and sedentary behaviour on health.

**Conclusions:**

These research priorities can help to guide decisions on future research directions.

## Background

Recent research has shown that both physical activity and sedentary behaviour are associated with a wide range of current and future health outcomes [1-3]. In fact, physical activity and sedentary behaviour are two independent and not mutually exclusive behaviours with different effects on health outcomes [4]. In the short term, physical activity has been shown to be moderately and positively associated with bone health, aerobic fitness, blood lipid levels, self-esteem, mental activity and fundamental movement skills in children and adolescents [1-3,5]. In the long term, both physical activity and sedentary behaviour have been identified as major, independent, modifiable risk factors for mortality and morbidity from many chronic, non-communicable and potentially preventable diseases [6-9]. New evidence also suggests that the relation between sedentary behaviour and all-cause end cardiovascular disease mortality is independent of physical activity levels [7].

Chronic diseases place a large economic burden on health services and impose significant costs on society (e.g. premature death, underappreciated economic effects and greater reliance on treatment) [8]. Although the ill effects of chronic disease largely manifest in adulthood, it is increasingly understood that the development typically begins in childhood or adolescence [9]. Therefore, physical activity levels and sedentary behaviour performed in the early years could potentially influence the development of disease later on in life.

At present, a large quantity of research is being conducted into the physical activity and sedentary behaviour of children, yet the research community remains challenged to provide a solid evidence base [10]. This is in part due to a lack of international research collaboration and a high degree of study repetition. The aim of this study therefore was to arrive at a set of international research priorities for physical activity and sedentary behaviour to guide more meaningful and focussed research. Specifically, this study aimed to answer the following research question: “What are the most important international research issues for the next 10 years in child and adolescent physical activity and sedentary behaviour?” Agreement on research priorities may help to inform evidence-based policy, guide funding allocation, and direct research options for postgraduate students [11,12].

### Existing literature

To identify existing evidence in this area, a systematic review of the English and non-English literature was performed using the following search terms: physical activit* OR motor activity (MeSH) OR sedentary behavio* AND child* OR adolescen* OR youth* AND research priorit* OR research agenda* OR research issue*. The databases PsychINFO (1887–), SPORTDiscus (1949–), Cochrane (1992–), CINAHL (1937–), ERIC (1966–) and PubMed (1950–) were searched in May 2012. Additional studies were also identified by contacting experts, Google searching and identifying potential studies in the reference lists of identified studies. Only four previously published papers that arrived at research priorities in child physical activity and/or sedentary behaviour were identified [11,13-15]. A working paper by Bull et al. [11] identified research priorities in physical activity with a focus on low to middle income countries. Evenson and Mota [13] highlighted research on the determinants and outcomes of physical activity and made recommendations for future study designs. Mountjoy et al. [15] identified existing gaps in physical activity research for children, with a focus on the need for greater collaboration between sport and existing programmes. The final study by Fulton et al. [14] had two aims. Firstly, the study aimed to review the current knowledge of existing methods for assessing physical activity and sedentary behaviour. Secondly, on the basis of this, the study aimed to set research priorities on the use of reliable and valid measurement tools to assess physical activity and sedentary behaviour in children aged 2–5 years.

While these studies were valuable contributions, they also had many limitations, including unsystematic participant selection, unstructured data collection procedures, and limited reporting on the process followed to arrive at the research priorities. Furthermore, the participants involved in the decision-making processes did not always represent the broader community of researchers, either from a geographical or institutional point of view. In addition, the anonymity of participants was not maintained during the consensus process. These limitations warranted a further study with an aim to arrive at a set of research priorities by employing a structured and rigorous methodology and improving reporting quality.

## Methodology

Ethical approval for all aspects of the methodology was granted by the University of South Australia Human Research Ethics Committee in September 2011.

This study employed a Delphi procedure. This procedure is appropriate for research questions which cannot be answered with complete certainty, but rather by the subjective opinion of a collective group of informed experts [16]. It allowed systematic refinement of the experts’ opinions over the course of several rounds while minimising confounding factors present in other group response methods [17-20].

The experts who participated in the Delphi procedure were identified by a 3–step procedure. Firstly, the lead study investigators independently recommended known researchers for the study. Secondly, a lengthy and extensive search was carried out to identify potential researchers from every world region and sub-region. Identifying potential experts from these regions involved searching for staff of relevant international bodies, government departments, non-government organisations, professional organisations and educational institutions. Thirdly, following email communication with the experts who have previously been identified, new experts were referred to the study investigators.

Once participants had been identified, it was important to determine their eligibility for inclusion in the study. Thus they were assessed using pre-determined inclusion and exclusion criteria. To be eligible, a researcher had to be an author of at least one peer-reviewed scientific publication on the physical activity or sedentary behaviour of children or adolescents, and must hold (at the time of selection) a senior position in their organisation. In addition, the experts were deliberately chosen to give geographical coverage of every world region and sub-region. Relevant information was gathered from staff homepages, Scopus author searches, the Journal and Author Name Estimator (http://www.biosemantics.org/jane/) and other relevant Internet searches to ascertain whether a researcher met these criteria.

**Figure 1:**
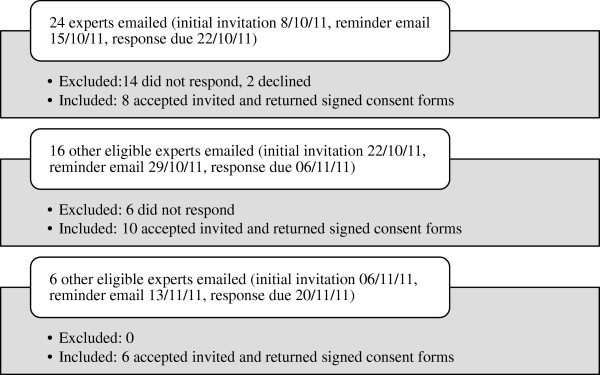
Purposive sampling process undertaken.

Forty-six eligible experts were invited to participate, with each sent information and consent forms via email. As a whole, these participants were representative of every region and sub-region. Of those invited, 20 did not respond to the invitation, two declined to participate, and 24 returned signed consent forms. An outline of this process is illustrated in Figure 1.

The 24 participating experts (17 male and 7 female) were randomly allocated to either Panel A or Panel B and assigned identification code names accordingly. Furthermore the following major institution types were represented by the selected experts; educational institutions, government organisations, non-government organisations, professional organisations and community organisations.

The Delphi procedure used three rounds [21], each consisting of data collection, data analysis and controlled feedback. The survey was administered entirely online using a *Survey Gizmo* questionnaire. A novel feature of this study was the use of two parallel panels of experts. The existence of an alternate panel was only made known to the participants in Round 3, when each panel was asked to rank the priorities of the other panel. This allowed quantitative comparisons to be made between each panel’s rankings of each research issue and cross-validated the rankings of research priorities developed by each panel.

To commence each round, experts were sent an email containing a direct link to the online questionnaire. Briefly, Round 1 required each expert to answer the question “What are the five most important research issues for the next 10 years in the area of child and adolescent physical activity and sedentary behaviour?” Each expert put forward five research issues which they believed were priorities in the area. They also provided a brief description of each issue and reasons why they believed the issue to be a priority. The three study investigators reviewed all issues that were provided by each panel, with common issues combined into a single issue. The experts were then fed back their panel’s list of research issues and asked to ensure that the five research issues they provided were accurately represented.

Round 2 then asked experts to “review the research issues put forward in Round 1 and rate how important they believe each issue is for global research in child and adolescent physical activity and sedentary behaviour”. Experts rated each research issue independently using a 5-point Likert scale (5 = very important, 4 = important, 3 = moderately important, 2 = of little importance and 1 = unimportant). The three study investigators then short-listed each panel’s research issues to 20 according to those with highest mean Likert scale ratings. Following this, the top 20 research issues from each panel were fed back to the experts of the relevant panels.

In Round 3, experts were first asked to “rank their panel’s top 20 research issues in order of perceived international importance in child and adolescent physical activity and sedentary behaviour over the next 10 years”. The experts were then similarly asked to rank the alternate panel’s top 20 research priorities. The data analysis procedure was as follows. Firstly, the overall sum of each panel’s rankings was calculated for Panel A and Panel B’s top 20 research issues. Secondly, the two lists of research issues were combined with common issues provided by both panels merged. This resulted in 29 unique issues. Thirdly, the experts’ individual rankings for each research issue were summed. This allowed the issues to be ranked according to the sum of Panel A and Panel B’s overall rankings for each issue. Intra-panel agreement was quantified using Spearman’s rho by creating a matrix to compare individuals’ rankings to one another within the same panel. Inter-panel agreement was also quantified using Spearman’s rho to compare the overall sum and rank for each issue between panels.

## Results

### Expert demographics

All 24 experts completed the three Delphi rounds. Data was collected on the 24 experts’ geographical distributions, institutional affiliations and years worked in the study area.

**Figure 2:**
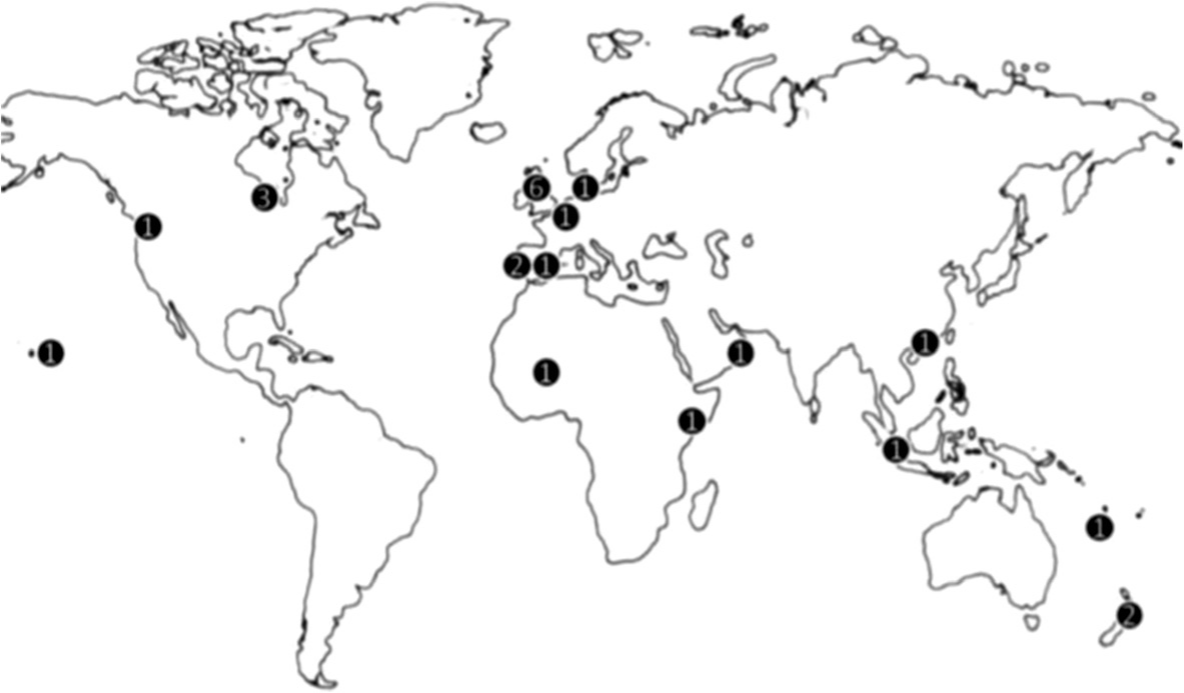
**Geographical distributions of participating experts.** The numbers indicate the number of participating experts from that region.

As a group, the 24 experts represented every geographical region and 12 sub-regions. This geographical distribution is illustrated in Figure 2.

In terms of institutional affiliation, twenty-three experts acknowledged they were affiliated with an educational institution, eleven were affiliated with a professional organisation, six with an international organisation, six with a non-government organisation and four with a government organisation. It was noted that due to the nature of their work, experts were often affiliated with more than one institution type.

In regards to years worked in the study area, twelve experts had worked in for greater than 16 years, five had worked for 11 to 15 years, four had worked for 6 to10 years and three had worked for less than five years.

### Results from Delphi rounds

In Round 1, each expert put forward five research issues. Collectively this provided a total of 120 issues across all 24 experts, with 60 for each panel. Following qualitative reduction of overlapping issues, 26 issues from Panel A and 34 issues from Panel B, were carried forward to Round 2. On reviewing the amended list, all exerts agreed that the issues they had raised were adequately represented.

From Round 2, the mean Likert-scale ratings were used to determine the top 20 issues for each panel. For Panel A, the mean Likert-scale ratings of the top 20 issues ranged from 3.5 to 5.0, with 18 of 20 issues having a median rating of >4.0 (“important”). For Panel B, the mean Likert-scale ratings of the top 20 issues ranged from 4.0 to 4.8, with all 20 research issues having a median rating of >4.0.

In Round 3, the 20 issues from Panel A and 20 issues from Panel B were qualitatively analysed to form one list. Eleven of each panel’s top 20 research issues were common to both panels and were therefore combined, with the remaining 18 issues (nine from each panel) unique. The resultant was a set of 29 unique research issues that were then ranked in order of importance by summing Panel A and Panel B’s rankings for each issue Table 1.

**Table 1:** Ranked set of 29 international research priorities in child and adolescent physical activity and sedentary behaviour

**Issue**	**Panel A sum**	**Panel B sum**	**Overall sum**	**Rank**
**Developing effective and sustainable interventions to increase children’s PA long-term.**	82	69	150	1
**Policy and/or environmental change and their influence on children’s PA and SB.**	84	81	165	2
**Prospective, longitudinal studies of the independent effects of PA and SB on health from birth to middle age**	89	98	186	3
The dose–response relationships between PA, SB and health	105	81	186	4
**SB's association with health outcomes, independent of other behaviours**	96	105	201	5
**Understanding the theory behind changing children’s activity levels and behaviours.**	105	99	204	6
How to create effective population-based interventions for the least active children.	107	108	215	7
Research on PA and SB in relation to obesity prevention	109	110	219	8
How to decrease child and adolescent screen time.	99	121	220	9
Cultural and parental practices related to PA and childrens’ behaviours.	122	99	221	10
Understanding the mediators and moderators of SB.	126	101	227	11
**Characterising different types of SB and then determining whether all SB is detrimental to children and adolescents’ health**	112	119	231	12
Effect of PA on cognitive function in youth.	100	137	237	13
Effects of the environment, on PA and SB.	117	123	240	14
**Effects of technology on SB.**	129	123	251	15
Tracking lifecourse changes in fitness and PA	127	134	261	16
**Improving objective measurement of children’s PA and SB**	118	147	265	17
Increasing children's active transportation.	140	139	279	18
Psychological and social factors associated with children and adolescents' PA and SB.	161	122	283	19
**Research into PA and SB on health in under five year olds.**	155	147	301	20
**Examination of the 24 hr patterns in children’s PA and SB in relation to health.**	153	154	307	21
Educating children on making better lifestyle choices regarding PA and SB	132	184	316	22
Determining the beneficial effects of mass advertising campaigns on PA.	155	175	330	23
The status of PA and SB at schools.	157	180	337	24
PE for health, quality of life and participation in the Culture of Movement	167	177	344	25
**Sport for enjoyment and participation and children's propensity to be active.**	178	169	347	26
The role of civil community institutions in promoting after school PA.	169	179	348	27
PE and resource availability.	172	193	365	28
Injury prevention among youths in sport	225	193	418	29

PA = physical activity; SB = sedentary behaviour. Research issues in bold are those which were put forward by both panels.

**Figure 3:**
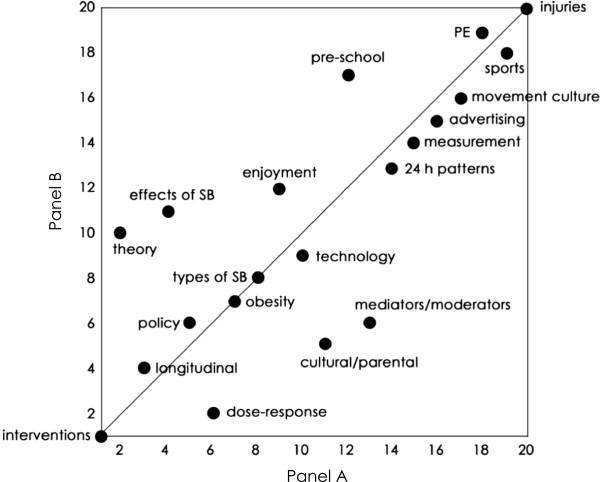
**Agreement between Panel A’s rankings and Panel B’s rankings of Panel A’s identified issues.** The line shown is the identity line.

**Figure 4:**
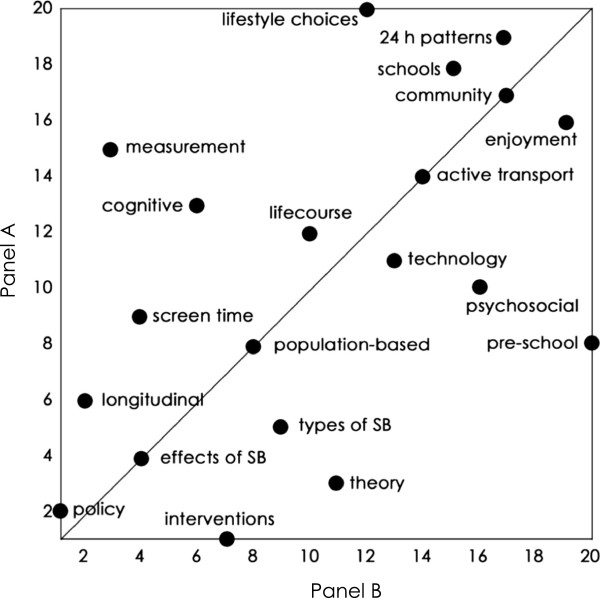
**Agreement between Panel B’s rankings and Panel A’s rankings of Panel B’s identified issues.** The line shown is the identity line.

There was only weak intra-panel agreement. The mean inter-individual *rho* (*±*95% CI) was 0.20 ±0.05 for Panel A and 0.13 ±0.04 for Panel B. The average standard deviation of the rankings for individual issues was 5.1 (Panel A) and 5.3 (Panel B). When Panel B ranked Panel A’s issues, the correlation was very strong (*rho ±*95% CI: 0.79 ±0.17), and when Panel A ranked Panel B’s issues, the correlation was strong (*rho ±*95% CI: 0.52 ±0.31). Figures 3 and 4 clearly illustrate the correlations for each research issue.

## Discussion

### Study outcomes

The primary outcome of this study was the development of 29 international research priorities in child and adolescent physical activity and sedentary behaviour. In order for the research priorities to be useful, it is important that they be neither too general nor too specific. The research priorities in this study appear broad enough to enable them to be transferable to researchers’ specific regions and contexts.

The final set of research priorities address a broad range of areas from epidemiology, determinants and correlates, through to intervention effectiveness and translational research. Of the 29 identified research priorities, ten related directly to translational research centred on intervention design and effectiveness. These focussed on specific behaviours (active transport, screen time, sport, physical education), settings (schools, communities, whole of population), or vehicles (mass advertising, policy). Translational research, centred on intervention design and effectiveness, can potentially guide governments and stakeholders to fund interventions that are the most effective, sustainable and transferable for changing behaviours [7]. This is important because to date, the research community has not been very successful at developing interventions for children and adolescents that bring about long-term and sustained change in health behaviours [10]. In addition, little attention has been given to the importance of the intervention setting and establishing what works in what situation and with whom [22].

Nine of the research priorities had a focus on capturing and quantifying the health benefits of engaging in physical activity and limiting sedentary behaviour, These research priorities were concerned with the impact of physical activity and sedentary behaviour on obesity, cognition, and general health and well being, and on describing behavioural patterns (across the day or the life-course or in specific populations such as pre-school children). Epidemiological research was considered important to address the cause, distribution and patterns of childhood physical activity and sedentary behaviour on current and future health [2,6,9,23].

Six research issues related to determinants and correlates research such as psychosocial and cultural/parental factors, the impact of technology, and the importance of enjoyment and lifestyle in general. Research that focuses on the determinants and correlates of behaviours is important. This is because while many correlates appear to be intuitively obvious, at present they have mixed support from high quality research [3].

Four issues did not fit into the aforementioned categories. They were related to the theory of behaviour change, injury prevention, measurement of behaviours and the physical education in culture of movement. Objective measurement of behaviours was ranked highly and is thought to be a “necessary first step for conducting meaningful epidemiological surveillance, public health research and intervention research” [14] p.124.

### Strengths and limitations

Unlike previously identified priority reports [11,13-15] this study employed a Delphi method to arrive at a more valid set of research priorities. Strengths related to the Delphi method include participant blinding, iterative data collection and controlled feedback between rounds. For example, the identities and responses of the experts were anonymised so that the identified research priorities could not be dominated by certain individuals [24]. Furthermore, the provision of controlled feedback allowed experts to individually consider their views in light of their panel’s collective opinion.

Other strengths related to the methodology were the use of criterion and purposive sampling methods. This procedure meant that all participants held a senior position in their respective organisations and had published in the study area. In addition, experts collectively represented every major world region and a wide range of discipline areas, affiliations and interests. This approach meant that the identified research issues were more likely to reflect the most important physical activity and sedentary behaviour issues facing the children and adolescents worldwide.

A novel component of this study was split-panel approach, which allowed comparisons to be made between the rankings given by the two expert panels. The experts from each panel were taken from the same population, given the same study information, answered identical online questionnaires and participated simultaneously and independently. One can therefore be confident that comparing the Round 3 rankings of Panel A and Panel B experts would provide valid measures of inter-panel agreement.

The weak intra-panel agreement was weak, which is likely a reflection of the natural variation of individual’s opinions and areas of interest within the broad study area. This weak agreement could also highlight the advantages of the methodology which retained anonymity and used an online mode of data collection. There were fewer pressures to conform to others opinions due to decreased likelihood of peer dominance and status. Evidence to reinforce confidence in the results is the strong to very strong (rho = 0.52–0.79) inter-panel agreement. While experts were invited from every United Nations sub-region (United Nations 2011), no experts from the following sub-regions took part: Southern Africa, Middle Africa, Caribbean, Eastern Europe, Australia, Central Asia and Western Asia. This was significant because many of these sub-regions are heavily involved in physical activity and sedentary behaviour research. Consequently, caution should be applied when recommending that the identified research priorities truly provide a global perspective. Nonetheless, these research priorities provide an international context from which priorities at the regional, national and local levels can be developed.

In addition the priorities were set for the broad area of child and adolescent physical activity and sedentary behaviour. Due to the generality of this topic, it may be that the research priorities are not relevant when conducting research into minority populations. For example, children and adolescents with disabilities may warrant different research issues not identified in this study.

### Implications for research

We hope that the identification of a set of ranked research priorities may contribute to more co-ordinated international research. For example, research priorities can help inform post-graduate students regarding where the current evidence gaps exist. This may be especially helpful for researchers who reside in less developed or marginalised research regions. In addition, encouraging more guided research can help to conceptualise how findings can be used as a basis for policy decisions. Lastly, research priorities can help to direct valuable funding into priority areas and away from studies on over-researched or lower priority topics.

## Conclusions

This study engaged two panels of study experts in a three-round Delphi communication procedure. The outcome of this procedure was the identification of a ranked set of 29 research priorities in child and adolescent physical activity and sedentary behaviour. For example, the top three ranked priorities were: developing effective and sustainable interventions to increase children’s physical activity long-term; policy and/or environmental change and their influence on children’s physical activity and sedentary behaviour; and prospective, longitudinal studies of the independent effects of physical activity and sedentary behaviour on health. We hope these research priorities will help inform the spectrum of future studies undertaken, guide post-graduate study choices, guide allocation of funding to priority areas and assist with policy decisions.

## Competing interests

The authors declare that they have no competing interests.

## Authors’ contributions

The study was conceived by GT and TO. LG was primarily responsible for conducting the participant selection process and the three rounds of data collection. LG, GT and TO were each involved in data analysis. LG produced the first draft of the paper with all other authors providing sections and critically reviewing the paper. All authors approved submission.
